# Impaired theta phase coupling underlies frontotemporal dysconnectivity in schizophrenia

**DOI:** 10.1093/brain/awaa035

**Published:** 2020-04-01

**Authors:** Rick A Adams, Daniel Bush, Fanfan Zheng, Sofie S Meyer, Raphael Kaplan, Stelios Orfanos, Tiago Reis Marques, Oliver D Howes, Neil Burgess

**Affiliations:** a1 Institute of Cognitive Neuroscience, University College London, 17 Queen Square, London, WC1N 3AZ, UK; a2 Division of Psychiatry, University College London, 149 Tottenham Court Road, London, W1T 7NF, UK; a3 Max Planck-UCL Centre for Computational Psychiatry and Ageing Research, 10-12 Russell Square, London, WC1B 5EH, UK; a4 Centre for Medical Image Computing, Department of Computer Science, University College London, Malet Place, London, WC1E 7JE, UK; a5 Wellcome Centre for Human Neuroimaging, University College London, 12 Queen Square, London, WC1N 3BG, UK; a6 Queen Square Institute of Neurology, University College London, London, WC1N 3BG, UK; a7 Brainnetome Center, Institute of Automation, Chinese Academy of Sciences, 95 Zhongguancun East Road, 100190 Beijing, China; a8 Kavli Institute for Systems Neuroscience, Norwegian University of Science and Technology, Trondheim, Norway; a9 South West London and St George’s Mental Health NHS Trust, Springfield University Hospital, 61 Glenburnie Rd, London SW17 7DJ, UK; a10 Department of Forensic and Neurodevelopmental Sciences, Institute of Psychiatry, Psychology, and Neuroscience, King’s College London, De Crespigny Park, Denmark Hill, London SE5 8AF, UK; a11 Institute of Clinical Sciences, Faculty of Medicine, Imperial College London, Hammersmith Hospital, London, W12 0NN, UK; a12 Department of Psychosis Studies, Institute of Psychiatry, Psychology and Neuroscience (IoPPN), King’s College London, London, SE5 8AF, UK

**Keywords:** schizophrenia, hippocampus, prefrontal cortex, theta, spatial memory

## Abstract

Frontotemporal dysconnectivity is a key pathology in schizophrenia. The specific nature of this dysconnectivity is unknown, but animal models imply dysfunctional theta phase coupling between hippocampus and medial prefrontal cortex (mPFC). We tested this hypothesis by examining neural dynamics in 18 participants with a schizophrenia diagnosis, both medicated and unmedicated; and 26 age, sex and IQ matched control subjects. All participants completed two tasks known to elicit hippocampal-prefrontal theta coupling: a spatial memory task (during magnetoencephalography) and a memory integration task. In addition, an overlapping group of 33 schizophrenia and 29 control subjects underwent PET to measure the availability of GABA_A_Rs expressing the α5 subunit (concentrated on hippocampal somatostatin interneurons). We demonstrate—in the spatial memory task, during memory recall—that theta power increases in left medial temporal lobe (mTL) are impaired in schizophrenia, as is theta phase coupling between mPFC and mTL. Importantly, the latter cannot be explained by theta power changes, head movement, antipsychotics, cannabis use, or IQ, and is not found in other frequency bands. Moreover, mPFC-mTL theta coupling correlated strongly with performance in controls, but not in subjects with schizophrenia, who were mildly impaired at the spatial memory task and no better than chance on the memory integration task. Finally, mTL regions showing reduced phase coupling in schizophrenia magnetoencephalography participants overlapped substantially with areas of diminished α5-GABA_A_R availability in the wider schizophrenia PET sample. These results indicate that mPFC-mTL dysconnectivity in schizophrenia is due to a loss of theta phase coupling, and imply α5-GABA_A_Rs (and the cells that express them) have a role in this process.

## Teaser

Communication between the frontal and medial temporal lobes is impaired in schizophrenia. Adams *et al*. find that when retrieving memories, subjects with schizophrenia have lower theta phase oscillatory coupling between these areas, and lower α-GABA_A_ receptor availability in the hippocampal area where coupling is lost.

## Introduction

Schizophrenia—characterized by delusions, hallucinations, and motivational and cognitive impairments—has long been hypothesized to be a disorder of functional brain connectivity (Friston, 1999). Dysconnectivity between the left medial temporal lobe (mTL)—in particular, the hippocampus—and prefrontal cortex is heavily implicated in the disorder (Weinberger *et al.*, 1992; Ellison-Wright and Bullmore, 2009).

Most direct projections from anterior hippocampus to prefrontal cortex in humans (Croxson *et al.*, 2005) and non-human primates (Barbas and Blatt, 1995) terminate in medial prefrontal cortex (mPFC). This direct, unidirectional hippocampal-mPFC projection is heavily implicated in functions relevant to psychiatric disorders, e.g. working memory, context-dependent memory, planning, decision-making, and contextual regulation of fear (Godsil *et al.*, 2013). Moreover, both participants with a diagnosis of schizophrenia and their unaffected relatives are substantially impaired at spatial working memory tasks (Park *et al.*, 1995; Fleming *et al.*, 1997; Glahn *et al.*, 2003).

Several rodent (Colgin, 2011) and human (Anderson *et al.*, 2010; Guitart-Masip *et al.*, 2013) electrophysiology studies have shown that functional hippocampal-PFC coupling is strongest in the theta band (∼4–10 Hz in rodents, ∼1–8 Hz in humans) (Jacobs, 2014). Crucially, the strength of theta phase coupling between these regions in rodents also correlates with memory performance for spatial, contextual and reward contingency associations (Jones and Wilson, 2005; Benchenane *et al.*, 2010; Hyman *et al.*, 2010; Kim *et al.*, 2011).

Hippocampal theta is generated by medial septal projections and local interactions between pyramidal cells and interneurons (Colgin, 2013). Oriens-lacunosum/moleculare (OLM) cells (a type of hippocampal interneuron) express somatostatin and fire spontaneously in the theta range, unlike parvalbumin-positive interneurons (Huh *et al.*, 2016). Inhibiting somatostatin (but not parvalbumin) interneurons in mPFC during encoding impairs spatial working memory and also hippocampal-mPFC theta synchrony (Abbas *et al.*, 2018). Importantly, Magnin *et al.* (2019) showed that somatostatin (but not parvalbumin) interneurons in CA1 express γ-aminobutyric acid type A receptors containing the α5 subunit (α5-GABA_A_Rs)—these are abundant in hippocampus and mPFC (Burt *et al.*, 2018)—and that α5-GABA_A_Rs modulate spatial memory. Moreover, subjects with schizophrenia show greater post-mortem reductions in somatostatin interneurons than other kinds (at least in the dorsolateral prefrontal cortex) (Fung *et al.*, 2010), and a deficit in GABA release in mTL (especially if antipsychotic-naïve) (Frankle *et al.*, 2015).

Intriguingly, rodent models of schizophrenia [*Df*(*16*)*A^+/–^* and maternal immune activation (MIA)] exhibit impaired hippocampal-mPFC theta coupling (Dickerson *et al.*, 2010; Sigurdsson *et al.*, 2010). Crucially, these frequency-specific changes can potentially be linked to biological mechanisms (Uhlhaas and Singer, 2010; Colgin, 2013; Hunt *et al.*, 2017) and thus drug targets. The *Df*(*16*)*A^+/–^* mouse models the 22q11.2 microdeletion in humans that causes schizophrenia in ∼30% of cases (Karayiorgou *et al.*, 2010). During memory guided decision-making, these animals show decreased hippocampal-mPFC theta coherence, and pretraining theta coherence correlates with subsequent learning speed (Sigurdsson *et al.*, 2010). Similarly, MIA rats—modelling prenatal infection, an environmental risk factor for schizophrenia—have lower hippocampal-mPFC coherence in theta (and other bands), and reduced phase locking of mPFC neuron firing to hippocampal theta (Dickerson *et al.*, 2010). Whether similar deficits in task-related theta coupling between mPFC and hippocampus exist in schizophrenia, however, is currently unknown, as functional MRI and EEG lack the temporal and spatial resolution, respectively to determine this, and no such magnetoencephalography (MEG) study has yet (to our knowledge) been performed.

Here, we addressed this question in participants with diagnoses of schizophrenia, almost half of whom were unmedicated. Control subjects were matched for age, gender and IQ. Our participants performed two tasks, chosen because they are both known to elicit hippocampal-mPFC theta coupling in humans. First, during MEG, participants performed a spatial memory task in which the cued retrieval of object locations has been shown to increase mPFC-mTL theta phase coupling (Kaplan *et al.*, 2014). Second, outside the scanner, participants performed a memory integration task in which the (hippocampal-dependent) (Bunsey and Eichenbaum, 1996) inference of unseen associations (i.e. A-C) between overlapping, observed pairs (i.e. A-B, B-C) is associated with increased mPFC-mTL theta phase coupling during encoding (Backus *et al.*, 2016) and hippocampal-mPFC blood oxygen level-dependent correlations during retrieval (Zeithamova and Preston, 2010). Given the potential importance of somatostatin interneurons in mediating theta coherence, we also measured α5-GABA_A_R availability (expressed on somatostatin interneurons) in a partly overlapping set of participants with schizophrenia and control subjects using ^11^C-Ro15-4513 PET—reported in full elsewhere (Marques *et al.*, 2020). ^11^C-Ro15-4513 is an inverse agonist and has ∼10 times the affinity for α5-GABA_A_Rs than for other GABA_A_Rs such as α1-GABA_A_Rs (Huang *et al.*, 2000) (most commonly expressed on parvalbumin interneurons in sensory areas) (Burt *et al.*, 2018).

To summarize, we predicted that subjects with schizophrenia would exhibit decreased mPFC-mTL theta phase coupling during cued spatial memory retrieval, and that subjects with schizophrenia would be impaired during spatial memory retrieval and memory inference.

## Methods and materials

### Participants

The study was approved by an NHS research ethics board (REF: 17/LO/0027). All participants gave written informed consent and were compensated for their time. We recruited 18 participants (15 male, three female) who met DSM-IV criteria for schizophrenia from local NHS trusts (11 from early psychosis services). We recruited 35 healthy control subjects (via UCL subject databases and local advertising), matching their age, sex and IQ (at group level) to the schizophrenia group.

General exclusion criteria were: current anticonvulsant or benzodiazepine use, metal in the head or upper body, age >60 years, poor vision even when corrected, and not having been educated in English (for IQ estimation). Control subjects were excluded if they had any history of neurological or psychiatric illness, and subjects with schizophrenia if they had another psychiatric diagnosis, including affective psychosis. Diagnoses were determined by the Structured Clinical Interview for DSM-IV-TR axis I disorders (SCID) (First *et al.*, 2007). Participants were asked not to smoke or consume caffeinated drinks on the day of testing.

We recorded the age, sex, handedness, years of education, IQ (Wechsler Test of Adult Reading, WTAR) and Digit Span (forward and backward) of all participants. In subjects with schizophrenia, we also recorded diagnosis, Positive and Negative Symptoms Scale (PANSS) (Kay *et al.*, 1987), antipsychotic medication, and a saliva cannabis test (Supplementary Table 1). Nine controls’ MEG scans were unusable: four had large artefacts, three suffered nausea and stopped, one fell asleep, and in one subject the fiducial markers fell off: leaving 26 controls (19 male, seven female). All schizophrenia participants’ MEG data were usable. One schizophrenia subject did not perform the memory integration task.

### Behavioural tasks

**Figure 1:**
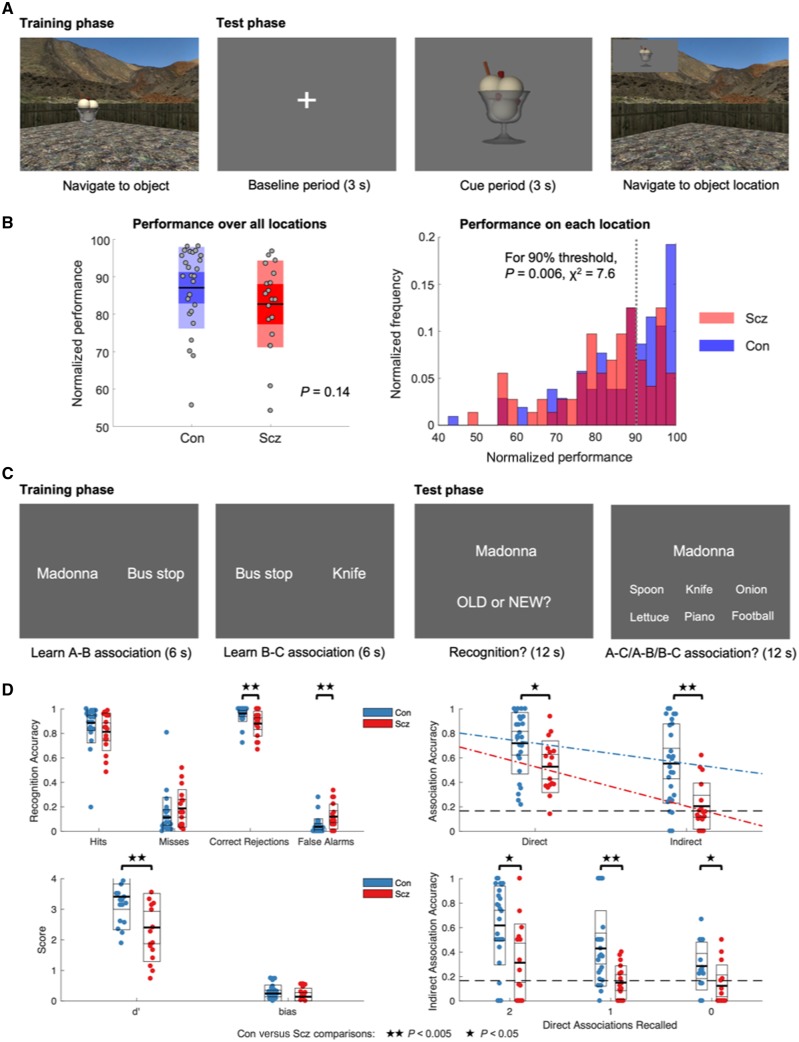
**Task structure and behavioural findings.** (**A**) The spatial memory task. In the training phase, participants navigate around the environment using a keypad and must remember the locations of four objects, only one of which is visible in each trial. In the test phase, the subject fixates a grey screen before being shown an image of one object (the cue period). They must then navigate to the remembered location of that object, starting from a random point, and press a button, at which point the object appears in its actual location to provide feedback on their performance. During their navigation to the location, the object is displayed at the top left corner of the screen, to ensure participants don’t forget what they are trying to locate. The next test trial starts when they ‘run over’ the visible object. (**B**) *Left*: Individual schizophrenia (Scz) participants’ average performance across all objects was numerically but not significantly worse than controls’ (Con). *Right*: A histogram showing the (normalized) frequency of average performance centiles for each object location (averaged across three sessions) for subjects with schizophrenia and controls, using translucent bars. Achievement of near ceiling performance is more common in controls than schizophrenia subjects—the proportion of subjects with schizophrenia above the 90th centile of performance was significantly lower than the proportion of controls (*χ*^2^ = 7.6, *P *=* *0.006): this comparison was also significant at other high performance thresholds (e.g. 80th or 85th centiles). (**C**) The memory integration task. During the training phase, participants were first asked to passively view successive pairs of stimuli displayed as text on screen and imagine those stimuli interacting as vividly as possible. Unknown to participants, they viewed two overlapping pairs (e.g. A-B, B-C) on non-consecutive trials that were drawn from a series of 12 events comprising a famous person, location and object. In the test phase, participants were first asked if each stimulus (randomly interleaved with an equal number of novel stimuli) had been presented earlier (testing recognition memory), and if it had been, which other stimulus it had been associated with. Importantly, some associations had been directly experienced (e.g. A-B, B-C) and some had to be inferred (e.g. A-C). (**D**) Performance on the memory integration task. *Top left*: Hits, misses, correct rejections and false alarms in the recognition memory test. *Bottom left*: d’ (or sensitivity) and response bias. *Top right*: Accuracy on direct and indirect associations, with best fit lines across conditions. *Bottom right*: Accuracy on indirect associations split by the number of direct associations from that event that the subject answered correctly (i.e. if a subject correctly recalled the direct association ‘Madonna-Bus stop’ but not ‘Bus stop-Knife’, their performance on indirect association ‘Madonna-Knife’ would contribute to the ‘1’ bar). The schizophrenia group are impaired (relative to controls) at indirect associations even when both direct associations have been recalled correctly. This indicates their reduction in indirect accuracy does not simply result from schizophrenia subjects remembering fewer direct associations. *P < 0.05; **P < 0.005.

The spatial memory task was first used by Doeller *et al.* (2008) and our version was based on an adaptation of that task by Kaplan *et al.* (2014). During the task, participants were asked to encode and retrieve the locations of different objects within a series of desktop virtual reality environments (Fig. 1A). The task was created using the Unity game engine (Unity Technologies Ltd). Each virtual reality environment was a flat 100 m sided square arena with a textured ground surface surrounded by a boundary wall and external landmarks projected at infinity. To locate oneself, orientation cues from distant landmarks must be combined with proximity to the boundaries.

In the training phase, participants navigate around the environment using a keypad and must remember the locations of four objects (per session), only one of which is visible in each trial. When the participant sees the object, they must approach and collide with (run over) it and then look around to try to encode their location; at this point the current object disappears and a new object appears. All four objects are seen twice each during the training phase.

In the test phase, the subject fixates a grey screen for 3 s (the baseline period) before being shown an image of one object for 3 s (the cue period). They must then navigate to the remembered location of that object, starting from a random point, and press a button, at which point the object appears in its actual location to provide feedback on their performance. The next test trial starts when they navigate to and collide with the visible object. Object locations were drawn pseudorandomly from 16 possible locations: each block contained an object near a corner, one near the middle of a boundary, and two closer to the middle of the space.

Participants completed up to three task sessions that made use of different objects and environments with up to eight test trials for each object in each session, providing a maximum of 32 test trials per session and 96 test trials in total for analysis. Each session lasted 15–30 min, depending on the subject’s speed, with short breaks in between sessions. Some participants did not complete all 32 trials in each session as MEG recording duration was capped at 30 min.

Performance was measured as the distance between the response location and true location of the object, normalized as the percentage of all possible distance errors for that object that were greater than the observed distance error, to account for the fact that objects near boundaries could produce larger errors than those near the centre (Miller *et al.*, 2018).

The memory integration task (Fig. 1C) was adapted from a version used previously (Horner and Burgess, 2013). During the training phase, participants passively viewed pairs of stimuli displayed as white text on a grey background for 6 s, with a 2-s intertrial interval. Participants were instructed to remember these associations by imagining the stimuli interacting as vividly as possible. Unknown to participants, they viewed two overlapping pairs (i.e. A-B, B-C)—on non-consecutive trials—drawn from 12 ‘events’ that comprised a famous person, object and location.

In the test phase, participants were presented with each stimulus, interleaved with 36 new stimuli, and asked whether they had appeared during training (OLD) or not (NEW)—testing recognition memory. Irrespective of their answer, if the stimulus had been presented in the training phase, they were then asked to select which of six stimuli it had been paired with. Importantly, this could test either directly viewed (i.e. A-B, B-C) or indirectly inferred (i.e. A-C) associations. Participants were given 12 s to respond to each question. They performed two blocks, each with a training phase of 24 encoding pairs and a test phase of 54 recognition questions, of which 36 were OLD items, of which 12 were tested on indirect associations. Thus participants were tested on 48 direct and 24 indirect associations in total, lasting ∼30 min including a break. Participants were instructed that they would find the test phase difficult and should make their best guess if they didn’t remember. They were not told that some associations would be indirect.

### MEG acquisition, preprocessing and analysis

MEG recordings were made using a 275-channel axial gradiometer system (CTF Omega, VSM MedTech) sampling at 480 Hz while participants sat upright in a magnetically shielded room. Head position coils were attached to nasion and left and right pre-auricular sites for anatomical co-registration. Eye movements were recorded using an Eyelink 1000 eye tracker (SR Research). The data were imported into SPM12 (Litvak *et al.*, 2011) (Wellcome Centre for Human Neuroimaging, London, UK) and downsampled to 200 Hz. Eye blink and heartbeat artefacts were identified by ICA and removed using FieldTrip (Oostenveld *et al.*, 2011) and EEGLAB (Delorme and Makeig, 2004). High-pass (1 Hz) and notch (48–52 Hz) filters were applied and the data were epoched and merged across sessions. All SPM images were smoothed using a 12 × 12 × 12 mm full-width at half-maximum Gaussian kernel. Trials containing signal artefacts (on average, 14/90 control and 20/88 schizophrenia trials) were identified and removed by visual inspection, resulting in 76 [standard deviation (SD) = 15] trials per control and 68 (SD = 19) per schizophrenia subject.

MEG source localization was conducted using the linearly constrained minimum variance (LCMV) beamformer in SPM12 with a single-shell forward model to generate maps of the mean source power difference between conditions on a 5 mm grid (Barnes and Hillebrand, 2003) co-registered to MNI coordinates (Kaplan *et al.*, 2014). Theta phase in each voxel was extracted by applying the Hilbert transform to the 1–8 Hz band-pass filtered time series generated by the beamformer.

**Figure 2:**
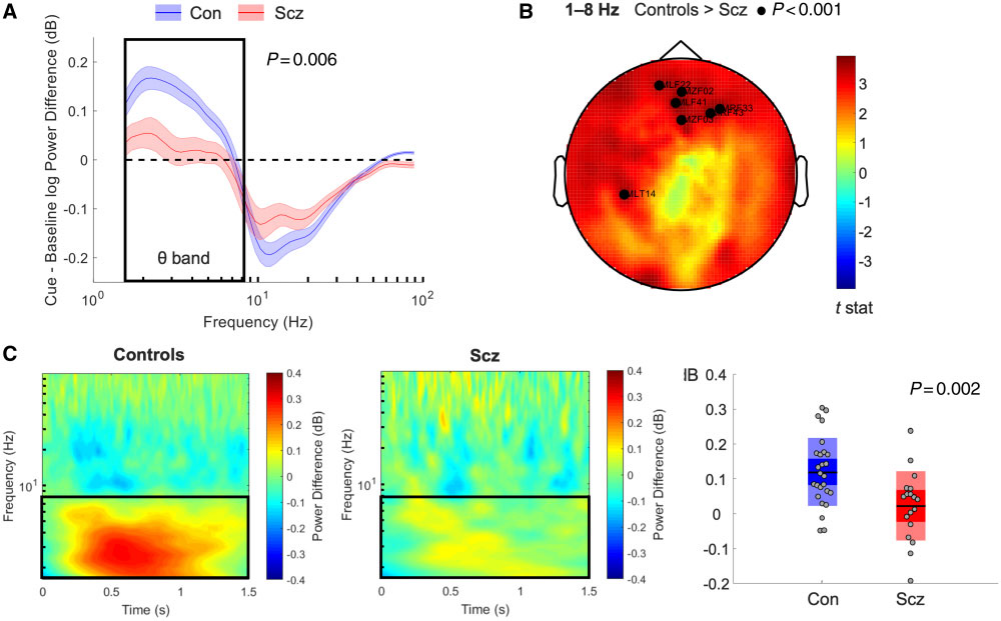
**Analysis of sensor-level power in the spatial memory task.** (**A**) Power changes, averaged across all sensors, between the first 1.5 s of the cue and the final 1.5 s of the baseline periods. Differences between groups were apparent at *P *<* *0.0125 (Bonferroni correction for four frequency bands) in the theta band (1–8 Hz, indicated with a box). (**B**) Scalp plot showing differences in the 1–8 Hz theta band between groups during the same period. Highlighted sensors show significant differences at *P *<* *0.001 (uncorrected). (**C**) *Left*: Time frequency plots showing power changes across the sensors highlighted in (**B**) during the same period following removal of cue period eye movement variance and the average event-related field across all trials. This illustrates the loss of 1–8 Hz theta power (black square) in schizophrenia (Scz) subjects during the cue period. *Right*: Average 1–8 Hz theta band power changes during the same period across participants following removal of the event-related field and eye movement regression.

We focused our analyses on a 1–8 Hz theta band because hippocampal theta occupies a broader frequency band than the traditional 3–7 Hz often used in cortical M/EEG studies. In particular, (i) invasively-recorded oscillations in human hippocampus appear to show both low (2–5 Hz) and high (6–9 Hz) theta bands (Bush *et al.*, 2017); (ii) those in the lower theta frequency range seem functionally equivalent to rodent theta (4–10 Hz) (Jacobs, 2014), given their relation to spatial memory encoding (Lega *et al.*, 2012) and retrieval (Watrous *et al.*, 2013; Bohbot *et al.*, 2017); and (iii) *Df*(*16*)*A^+/–^* mice show abnormal hippocampal-mPFC coupling in both the 1–4 Hz and 4–6 Hz but not the 7–10 Hz ranges. In any case, our group differences were clearly not confined to the 3–7 Hz range (Fig. 2).

All MEG analyses described here compared the first 1.5 s of the cue period with the last 1.5 s of the preceding fixation period (Fig. 2C). As both event-related fields and/or eye movements evoked by the visual cue can confound this comparison, we attempted to eliminate their effects in key analyses. First, the event-related field was removed by subtracting the average waveform across trials from single trial waveforms prior to power analyses. Second, any effect of the eye movement signal identified by ICA was removed by linear regression across trials, with subsequent power and coupling analyses performed on the residual values (Kaplan *et al.*, 2014).

Theta coupling between voxels was estimated using the phase locking value (Lachaux *et al.*, 1999) (PLV), which is defined as the resultant vector length of phase differences between source and seed voxels over time: a larger value indicating reduced variance in the phase difference (and therefore greater phase coupling) between two signals. In previous phase coupling analyses, the seed voxel was chosen to be that with the greatest theta power increase from baseline to cue within 20 mm of the group maximum (Kaplan *et al.*, 2014). Here, we did the same, using the overall average peak (after regressing out eye movements) of (8 52 8). This voxel is in the right medial part of Brodmann area (BA)10 (superior rostral gyrus), and the 20-mm radius encompasses parts of BA10, BA9 and BA32 on both sides. Given we had more control subjects than participants with schizophrenia, the overall average peak could potentially favour positive findings in controls, if it were closer to the average peak location of control subjects rather than participants with schizophrenia. This was not the case, however, as the subjects with schizophrenia average cue-related theta power increase [at (3 53 13)] was slightly closer to the overall average than the controls’ average [at (−2 53 9)]. We also preferred to use the overall average peak rather than group-specific peaks, because (i) the control’s average was much more inferior and thus closer to potential eye-related theta power artefacts; (ii) the overall average was close to the (0 58 22) group peak found in controls in Kaplan *et al.* (2014); and (iii) we had no reason to suspect different projection sites in controls and subjects with schizophrenia, so it seemed best to pool the data to obtain the most accurate estimate.

Although PLV measures phase coherence, it can be biased by concurrent changes in power that affect the signal-to-noise ratio (Muthukumaraswamy and Singh, 2011). We sought to eliminate this potential confound by linear regression, removing any effect of trial-by-trial variance in (mean-corrected) power across baseline (i.e. fixation) and cue periods and then contrasting the residual PLV values (Kaplan *et al.*, 2014). We removed any linear effect of head movements in each trial on PLV values using the same method.

**Figure 3:**
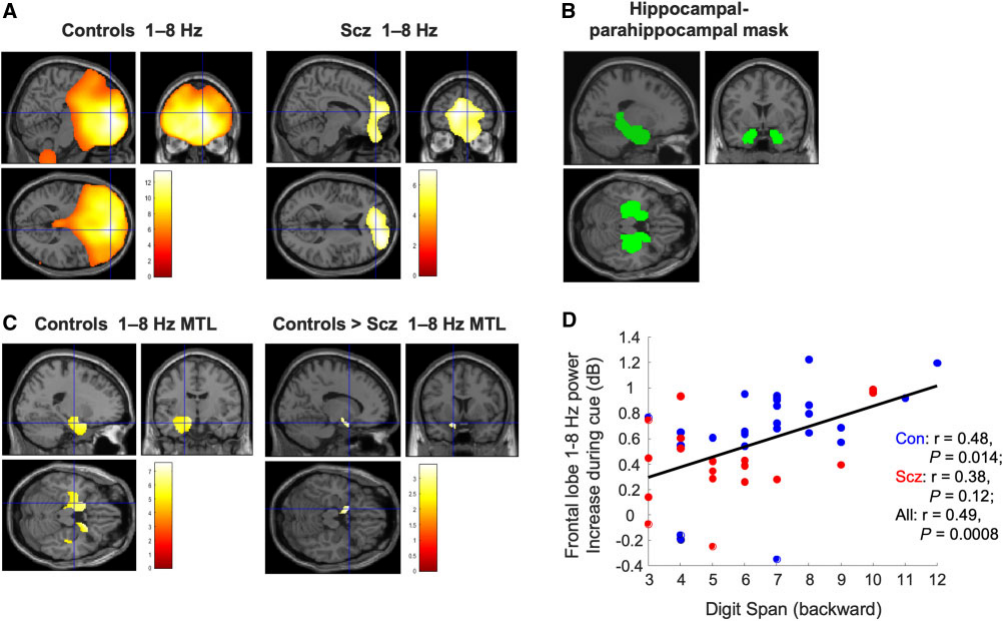
**Analysis of source localized power in the spatial memory task.** All results are overlaid on a canonical Montreal Neurological Institute T_1_ image. (**A**) Source localized 1–8 Hz theta power increases during the first 1.5 s of the cue period, compared to an immediately preceding 1.5 s baseline period, for controls and schizophrenia subjects (after regression of eye movement variance). Both groups exhibit significant increases centred on mPFC [controls: peak (14 54  −4), *Z *=* *7.18, *P*_FWE_ < 0.001; schizophrenia subjects: peak (22 58 12), *Z *=* *4.73, *P*_FWE_ = 0.003]. The crosshairs in each plot are at (8 52 8), the group peak and seed chosen for subsequent phase coupling analysis. Images are thresholded at *P*_FWE_ < 0.05. (**B**) The dilated bilateral hippocampal-parahippocampal gyrus (HC + PHG) mask (Maldjian *et al.*, 2003) used for all mTL small volume correction analyses. (**C**) Source localized 1–8 Hz theta power increases during the cue period within the HC + PHG mask. Controls showed a large increase in anterior mTL theta power bilaterally (following eye movement regression), particularly on the left [left peak at (−12 0  −12), *Z *=* *5.15, *P*_FWE(SVC)_ < 0.001, right peak (20 18  −34), *Z *=* *5.42, P_FWE(SVC)_ < 0.001]. Image is thresholded at *P*_FWE_ < 0.05. Subjects with schizophrenia showed a much smaller increase on the right side (not shown); and control subjects showed a significantly greater increase than schizophrenia subjects on the left [peak (−14 8 −18), *Z *=* *3.21, *P*_FWE(SVC)_ = 0.019]. The image is thresholded at *P*_unc_ < 0.005 for display purposes. (**D**) Relationship between frontal lobe cue-related 1–8 Hz power increase and performance on the more demanding (backward) condition of the Digit Span across participants.

To identify potential correlations between theta power or PLV and trial-by-trial performance, we extracted beta coefficients for the linear relationship between distance error (rather than the normalized centile measure of performance in Fig. 1B, as these distributions were very skewed) and power (or PLV) and then examined the distribution of beta coefficients in each voxel, across participants, using standard SPM analyses. All mTL SPM results are small volume corrected for a dilated (by one voxel in each direction) bilateral hippocampal and parahippocampal mask (Fig. 3B) generated using the WFU Pick Atlas toolbox (Maldjian *et al.*, 2003).

To compute between-subject correlations of behavioural performance and average (not trial-by-trial) cue-related power or PLV changes within a given area, we used SPM to calculate the eigenvariate (in this context, similar to the average) of power or PLV within a mask (defined by the WFU Pick Atlas or by an SPM contrast) for each subject.

### PET imaging

Six controls and 11 subjects with schizophrenia from the MEG study also underwent a PET scan using the radiotracer ^11^C-Ro15-4513 at the Invicro Centre for Imaging Sciences, London. They were a subsample from a larger PET study (29 controls and 31 schizophrenia subjects), whose protocol is reported in Marques *et al.* (2020). Briefly, all subjects were male and matched in age, and were not taking any psychotropic medication other than antipsychotics; 10 participants with schizophrenia were unmedicated. Dynamic PET scans were acquired over 90 min using a Siemens ECAT EXACT HR scanner (CTI/Siemens, model 962). During the PET acquisition, arterial blood data were sampled via the radial artery, in order to compute blood time-activity curves. High resolution T_1_-weighted volumes were also acquired using a 3 T MR scanner (Magneton Trio Syngo MR B13 Siemens 3T; Siemens AG), and dynamic PET data were corrected for inter-frame motion and aligned with the individual’s structural T_1_ MRI by minimizing a mutual information cost function (SPM8). A neuroanatomical atlas was co-registered to each subject’s image space (using MIAKAT, www.miakat.org) and applied to the dynamic PET data to derive regional time-activity curves. α5-GABA_A_R availability was indexed using the distribution volume (V_t_) of ^11^C-Ro15-4513. As previously (Myers *et al.*, 2012; Horder *et al.*, 2018), V_t_ was calculated using a metabolite-free arterial plasma input function and the two-tissue compartmental model (2TCM) solved with non-linear least squares at the regional level and the Logan graphical method at the voxel level.

### Data availability

The data that support the findings of this study are available from the corresponding author, upon reasonable request.

## Results

### Behavioural results

Schizophrenia and control groups performed two tasks known to elicit mPFC-hippocampal theta coupling: a spatial memory task during MEG and a memory integration task (without MEG). The groups were well-matched in age, sex and IQ (Supplementary Table 2), although the controls spent more years in education [15.9 versus 14.2, *t*(42) = 3.3, *P *=* *0.003] and, despite IQ matching, performed better on the more difficult backward condition of the digit span (6.8 versus 5.4, rank test *Z *=* *2.2, *P *=* *0.03).

On the spatial memory task, controls and participants with schizophrenia showed no difference in overall performance (rank test, *Z *=* *1.49, *P *=* *0.14; Fig. 1B, left plot), although fewer schizophrenia subjects than controls performed near ceiling (90th centile) levels for individual object locations (*χ*^2^ = 7.6, *P *=* *0.006; Fig. 1B, right plot). The right plot in Fig. 1B is a histogram showing the probability a participant’s average performance (across three sessions) on each of four object locations falls into the given percentile ranges of possible distance errors (see ‘Materials and methods’ section). Given the reasonable performance of the schizophrenia group, we could interpret the imaging results with confidence that they were performing the task similarly to the controls subjects.

Conversely, the schizophrenia group were significantly impaired at the memory integration task. During recognition, they showed increased false alarms [*t*(41 = 3.2, *P *=* *0.002, Hedges' *g *=* *1.0, 95% confidence interval (CI) 0.3–1.6; Fig. 1D] which, despite no increase in misses [*t*(41 = 1.5, *P *=* *0.16], resulted in lower *d*′ [*t*(41 = 3.0, *P *=* *0.006, Hedges' *g *=* *0.9, 95% CI 0.2–1.5; Fig. 1D]—i.e. less separation of ‘signal’ and ‘noise’ distributions. Importantly, this was unlikely to arise from an increased response bias (i.e. participants with schizophrenia always responding ‘OLD’), which was numerically lower than controls’ (Fig. 1D).

In addition, schizophrenia subjects showed impaired memory for both direct and inferred associations [main effect of group, *F*(1,41) = 12.4, *P *=* *0.001, *η^2^* = 0.23; Fig. 1D], but especially the latter: there was a group × association interaction [*F*(1,41 = 9.8, *P *=* *0.003, *η^2^* = 0.19] caused by a larger group difference in the inferred [*t*(41) = 4.0, *P *=* *0.0003, Hedges' *g *=* *1.2, 95% CI 0.5–1.9] than the direct association [*t*(41) = 2.6, *P *=* *0.013, Hedge’s *g *=* *0.8, 95% CI 0.1–1.4]. Indeed, despite IQ-matching, the schizophrenia group were no better than chance at inferring associations [*t*(16) = 0.8, *P *=* *0.4], whereas the control group were substantially better [*t*(25) = 6.2, *P *=* *10^−6^, Hedges' *g *=* *1.2, 95% CI 0.7–1.7]. Importantly, the schizophrenia group’s impairment at inferred associations was not simply due to poorer memory for direct associations: even if two direct associations (A-B, B-C) were recalled correctly, the schizophrenia group were still worse than the controls at inferring A-C [*t*(36) = 2.9, *P *=* *0.006, Hedges' *g *=* *0.9, 95% CI 0.2–1.6] and no better than chance [*t*(14) = 1.8, *P *=* *0.1; Fig. 1D].

### MEG power results

We examined task-related changes in theta power and phase coupling during cued spatial memory retrieval (Kaplan *et al.*, 2014). Specifically, we contrasted oscillatory power or coupling during the first 1.5 s of each cue period with the immediately preceding 1.5 s of the fixation period.

First, we found that participants with schizophrenia showed lower cue versus baseline theta power increases across all sensors [*t*(42) = 2.9, *P *=* *0.006, Hedges' *g *=* *0.9, 95% CI 0.2–1.5; Fig. 2A]. Conversely, there were no significant group differences in alpha (8–14 Hz), beta (15–30 Hz) or gamma (40–90 Hz) after Bonferroni correction. Subsequent analysis of power changes across the scalp revealed significant group differences in theta power located over frontotemporal and temporo-occipital sensors, respectively (Fig. 2B). Time-frequency plots, averaged over these groups of sensors, demonstrate that induced theta power increases were prominent throughout the first 1 s of the cue period in the control group, but much less prominent and sustained in the schizophrenia subjects (Fig. 2C). Importantly, theta power differences could not be accounted for by an event-related field evoked by the visual cue, or by more eye movement during the cue period (see ‘Materials and methods’ section).

Next, we attempted to localize the source of these cue-related theta power increases. We found that both controls and schizophrenia subjects showed robust 1–8 Hz theta power increases with maxima around mPFC [controls: peak (14 54  −4), *Z *=* *7.18, *P*_FWE_ < 0.001; schizophrenia: peak (22 58 12), *Z *=* *4.73, *P*_FWE =_ 0.003; Fig. 3A]. Using a dilated bilateral hippocampal and parahippocampal mask (Fig. 3B), we found that controls also showed a robust increase in 1–8 Hz theta power in bilateral mTL [left peak at (−12 0  −12), *Z *=* *5.15, *P*_FWE(SVC)_ < 0.001, right peak (20 18  −34), *Z *=* *5.42, *P*_FWE(SVC)_ < 0.001; Fig. 3C], whereas the schizophrenia group only showed a power increase on the right at a very low threshold [peak (30 12  −22), *Z *=* *2.47, *P*_unc_ = 0.007]. Importantly, the increase in 1–8 Hz theta power in left anterior mTL was significantly higher in controls than schizophrenia subjects [peak (−14 8  −18), *Z *=* *3.21, *P*_FWE(SVC)_ = 0.019; Fig. 3C).

We assessed whether the smaller increase in theta power during the cue period in the schizophrenia group could be attributed to greater power during the baseline period in that group. When comparing controls and schizophrenia subjects theta power during the baseline period only, no voxels within the mask were significant at *P*_unc_ < 0.005.

Finally, we asked whether there was any correlation between frontal or mTL theta power increases and performance on the spatial memory task. The controls’ robust mTL theta power increase did not correlate with trial-by-trial performance [*P*_unc_ > 0.005], and neither did mTL theta power in the schizophrenia group. Schizophrenia subjects’ performance weakly correlated with theta power in cingulate cortex only [peak (−6 22 24), *Z *=* *2.90, *P*_unc_ = 0.002]. Controls’ performance correlated with theta power in visual and parietal areas (not reported), but not cingulate cortex.

Interestingly, however, the frontal lobe theta power increase correlated with performance on the more demanding (backward) part of the Digit Span across all participants (*r *=* *0.49, *P *=* *0.008; Fig. 3D) and within controls (*r *=* *0.48, *P *=* *0.014) but was not statistically significant within schizophrenia subjects (*r *=* *0.38, *P *=* *0.12). However, frontal theta had no relationship with performance on the spatial memory task.

### MEG phase coupling results

**Figure 4:**
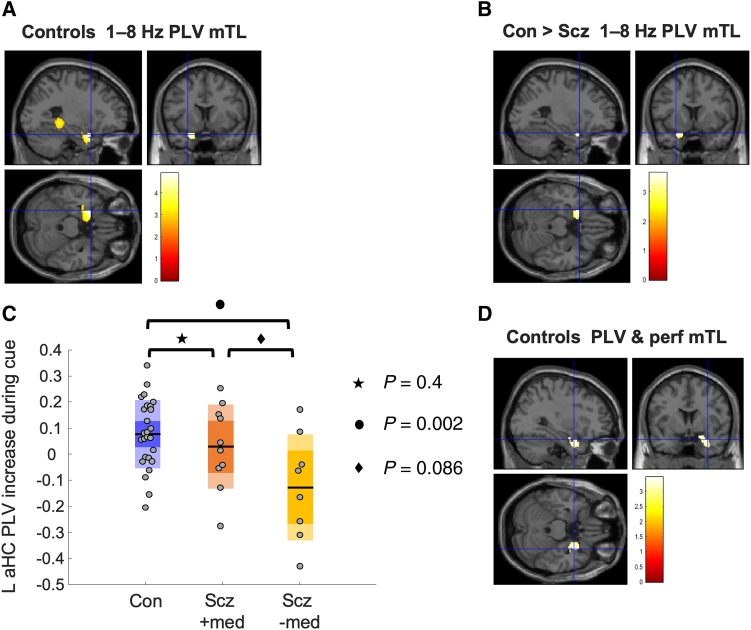
M**PFC theta phase coupling analysis.** All images are thresholded at *P*_unc_ < 0.005 for display purposes unless otherwise stated. (**A**) Controls showed significant increases in 1–8 Hz theta phase coupling (analysed using the PLV) during the cue period in two regions—one in posterior hippocampus [peak (−36  −42  −2), *Z *=* *3.51, *P*_FWE(SVC)_ = 0.018] and one in anterior mTL [peak (−26 10  −24), *Z *=* *4.07, *P*_FWE(SVC)_ = 0.003]. Subjects with schizophrenia did not show a significant theta increase in either mTL region. (**B**) Controls showed greater 1–8 Hz theta PLV than schizophrenia subjects in left anterior mTL, after correcting for trial-by-trial variance in both theta power and head movement and use of antipsychotic medication [peak (−26 10  −22), *Z *=* *3.37, *P*_FWE(SVC)_ = 0.024]. (**C**) Eigenvariates (similar to the average) of cue-related PLV increases in the significant region shown in (**B**) (after theta power regression). The schizophrenia group are divided according to antipsychotic medication use. It is clear that antipsychotics cannot be causing the schizophrenia group’s reduction in PLV as only the schizophrenia group OFF medication show a significant difference in PLV from control subjects (Hedges' *g *=* *1.3, 95% CI 0.4–2.2). The comparison between schizophrenia subjects ON and OFF medication was underpowered and not significant (Hedges' *g *=* *0.8, 95% CI −0.2–1.8). (**D**) Cue-related theta PLV increase in controls correlated positively with performance in right anterior mTL [peak (32 4  −24), *Z *=* *3.1, *P*_FWE(SVC)_ = 0.057, *P*_unc_ = 0.001]. Note: The colour bar scale corresponds to *t*-statistics in the SPM: here, of beta coefficients resulting from a linear regression of trial-wise performance on PLV on in each voxel.

Second, we examined changes in theta phase coupling between a subject-specific mPFC seed showing robust power increases during the cue period and the rest of the brain. In controls, two mTL regions showed robust PLV increases (Fig. 4A): left anterior mTL [peak (−26 10  −24), *Z *=* *4.07, *P*_FWE(SVC)_ = 0.003] and left posterior hippocampus [peak (−36 −44 −2), *Z *=* *3.51, *P*_FWE(SVC)_ = 0.018]. Conversely, in schizophrenia subjects, no areas within the mask were significant at *P*_unc_ < 0.005.

As predicted, we saw a loss of theta phase coupling with mPFC in schizophrenia subjects: the control versus schizophrenia group difference was significant in left anterior mTL [peak (−26 10  −22), *Z *=* *3.37, *P*_FWE(SVC)_ = 0.024; Fig. 4B], and was the strongest effect in the whole brain: nowhere else was significant at *P*_unc_ < 0.001. Moreover, this effect could not be accounted for by trial-by-trial variance in theta power, head movement or antipsychotic medication usage, each of which were included as nuisance regressors. There was also no significant difference in PLV in mTL between control subjects and schizophrenia subjects during only the baseline period [at *P*_unc_ < 0.005].

Interestingly, however, the difference in mPFC-mTL theta coupling between controls and schizophrenia subjects was weaker when nuisance regressors were omitted from this analysis [*P*_FWE(SVC)_ = 0.053]. The regressor contributing most to this change was antipsychotic medication. Figure 4C shows the eigenvariates (roughly, averages) of participants’ PLV (after theta power regression) within the left anterior mTL region that showed the peak PLV difference between controls and schizophrenia subjects (Fig. 4B). Only the comparison between controls and participants with schizophrenia OFF medication was significant [*t*(32) = 3.4, *P *=* *0.002, Hedges' *g *=* *1.3, 95% CI 0.4–2.2; controls versus schizophrenia ON medication *t*(34) = 0.9, *P *=* *0.4]. Schizophrenia subjects ON versus OFF medication did not show a significant difference in PLV [*t*(16) = 1.8, *P *=* *0.086, Hedges' *g *=* *0.8, 95% CI −0.2–1.8], although this comparison was clearly underpowered. Importantly, the overall difference in PLV between controls and all schizophrenia subjects (irrespective of medication status) is still large: Hedges' *g *=* *0.7 (95% CI 0.1–1.3). Very similar results were obtained without regression of theta power changes from the PLV.

**Figure 5:**
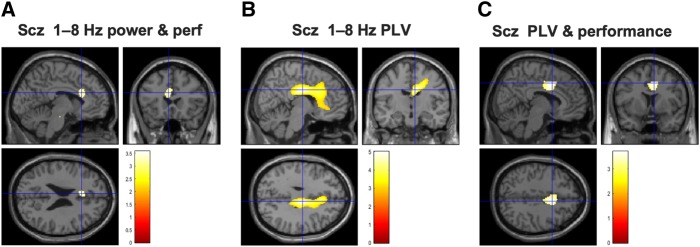
**Exploratory whole-brain analyses revealed theta power and phase coupling effects in a mid-cingulate region in schizophrenia subjects.** (**A**) In a whole-brain analysis, schizophrenia subjects’ (Scz) performance correlated with 1–8 Hz theta power in cingulate cortex only [peak (−6 22 24), *Z *=* *2.90, *P*_unc_ = 0.002]. The image is thresholded at *P*_unc_ = 0.005 for display purposes. (**B**) A whole-brain PLV analysis of the schizophrenia group found the most significant increase in a mid-cingulate area [peak (8  −12 30), *Z *=* *3.87, *P*_unc_ < 0.001], close to the cingulate area showing a theta power correlation to performance in subjects with schizophrenia. (**C**) In schizophrenia subjects, PLV increase and performance also correlated in mid-cingulate cortex [peak (6 2 38), *Z *=* *2.82, *P*_unc_ = 0.002]; the PLV-performance relationship here was stronger in schizophrenia subjects than in controls [peak (2 4 36), *Z *=* *2.81, *P*_unc_ = 0.002]. Subjects with schizophrenia may thus be using this node of the frontoparietal control network to compensate for their hippocampal-mPFC circuit dysfunction in this task.

We asked whether changes in mPFC theta phase coupling during the cue period correlated with task performance. In control subjects, PLV in right mTL increased with performance [peak (32 4  −24), *Z *=* *3.10, *P*_FWE(SVC)_ = 0.06, *P*_unc_ = 0.001; Fig. 4D]. This relationship fell far short of statistical significance in the schizophrenia group, however [peak (22  −10  −36), *Z *=* *1.87, *P*_unc_ = 0.031]. An exploratory analysis revealed the largest cortical PLV increase in schizophrenia subjects arose in a mid-cingulate area [peak (8  −12 30), *Z *=* *3.87, *P*_unc_ < 0.001], adjacent to the region showing a theta power correlation with performance (Fig. 5A and B). In schizophrenia subjects, across the whole brain, the strongest correlation with performance was in mid-cingulate cortex [peak (8 8 42), *Z *=* *3.13, *P*_unc_ = 0.001; Fig. 5C]: and this effect was stronger in schizophrenia subjects than in control subjects [peak (2 4 36), *Z *=* *2.81, *P*_unc_ = 0.002]. Although tentative, this may imply subjects with schizophrenia use the mid-cingulate region (part of the frontoparietal control network A) (Schaefer *et al.*, 2018) to compensate for hippocampal-mPFC circuit dysfunction.

We tested whether the schizophrenia group’s ability to increase mPFC-mTL theta phase coupling during the cue period correlated with task performance on the memory integration task. The measure of interest in the latter task was ‘direct condition performance – inferred condition performance’, as this isolates hippocampal function from overall memory impairment. In the schizophrenia group there was a negative relationship between this measure and cue-related PLV increase in the spatial memory task, but it didn’t reach significance (*r* = −0.41, *P *=* *0.10); there was no relationship between them in controls (*r *=* *0.03, *P *=* *0.9).

We assessed the specificity of mTL phase coupling differences to the theta band by repeating the analyses in the alpha (8–14 Hz), beta (15–30 Hz) and low gamma (40–60 Hz) bands (high gamma being hard to resolve at deep sources). There were no significant group PLV differences even at a liberal significance threshold [*P*_unc_ < 0.005], and the interaction of group × frequency was significant in the left mTL in a two-way ANOVA [peak (−10  −34 0), *Z *=* *3.16, *P*_unc_ = 0.001].

We assessed whether symptom severity rather than antipsychotic exposure could underlie the possible influence of medication on theta coupling, as symptoms were numerically but not significantly lower in subjects with schizophrenia ON medication (PANSS total mean = 46, SD = 10) than OFF medication (PANSS total mean = 57, SD = 23). PANSS and PLV did not correlate, however (*r* = −0.11, *P *=* *0.7; Supplementary Fig. 2). Cannabis use did not appear to affect PLV either: the PLV of the schizophrenia group testing negative (*n *=* *9) was almost identical to that of those testing positive (*n *=* *9) [*t*(16) = 0.02, *P *=* *1; Supplementary Fig. 3].

### PET results

**Figure 6:**
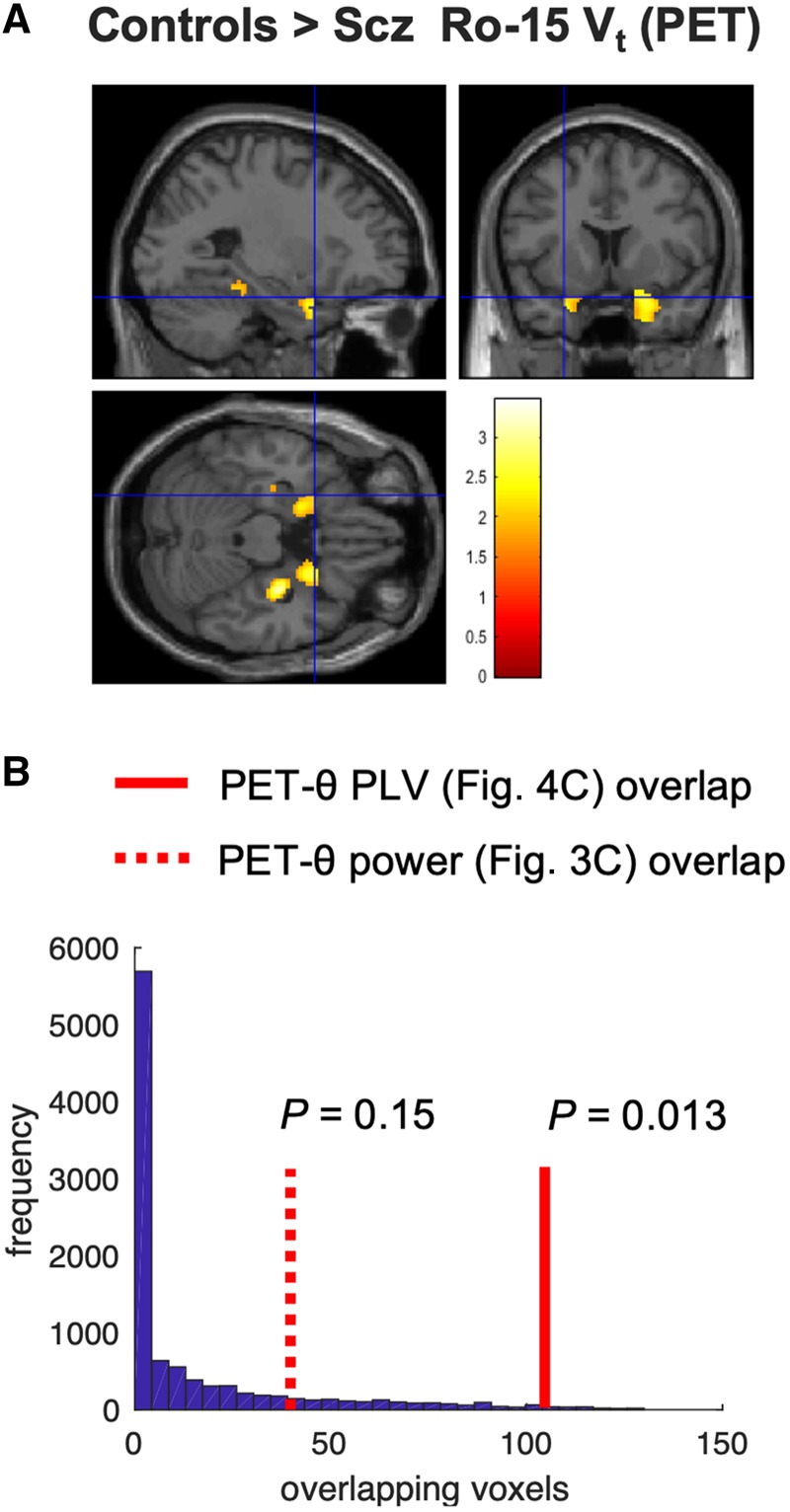
**PET analysis.** (**A**) A reanalysis of PET data presented by Marques *et al.* (2020), who found reduced α5-GABA_A_R availability in hippocampus in subjects with schizophrenia (Scz). We performed a voxel-wise analysis of these data to look for any overlap between areas of α5-GABA_A_R reduction and cue-related PLV decrease. At reduced threshold [*P*_unc_ < 0.05], the schizophrenia group had four areas of reduced α5-GABA_A_R availability >100 voxels in size: two in right anterior mTL [peak (32  −12  −20), *Z *=* *3.30, *P*_unc_ < 0.001; peak (28 18 −34), *Z *=* *2.75, *P*_unc_ = 0.003], one in left anterior mTL [peak (−20 4 −26), *Z *=* *2.40, *P*_unc_ = 0.008] and one in posterior hippocampus [peak (−20  −34  −12), *Z *=* *2.15, *P*_unc_ = 0.016]. The third (anterior mTL) area overlapped with the areas of reduced theta power (Fig. 3C, right) and PLV (Fig. 4B) in schizophrenia subjects: the crosshairs are placed at the peak contrast voxel from Fig. 4B. (**B**) The probabilities of the overlaps between the Ro-15 PET controls > schizophrenia contrast in (**A**) and the theta power and PLV controls > schizophrenia contrasts in Figs 3C and 4B were assessed using permutation testing (10 000 permutations). This histogram plots the number of overlapping voxels expected by chance: the theta PLV effect overlap is unlikely to be due to chance (*P *=* *0.013).

In an attempt to identify a potential molecular mechanism underlying the difference in mTL theta power and mPFC-mTL phase coupling between controls and subjects with schizophrenia, we turned to PET imaging of α5-GABA_A_Rs, found on hippocampal interneurons expressing somatostatin. Marques *et al.* (2020) found a significant reduction in α5-GABA_A_R availability in a hippocampal region of interest in an overlapping group of schizophrenia subjects (see ‘Materials and methods’ section); the hippocampus was the only region in the brain where α5-GABA_A_R availability was different in schizophrenia subjects versus controls. We performed a voxel-wise analysis of their whole sample (31 subjects with schizophrenia, 29 control subjects) to see if reduced α5-GABA_A_R availability in their schizophrenia group overlapped with mTL regions showing reduced theta power and PLV in our schizophrenia group. For this exploratory analysis, we used a permissive significance threshold [*P*_unc_ < 0.05] for the controls versus schizophrenia subjects α5-GABA_A_R binding contrast to see how widespread the α5-GABA_A_R reduction might be. Within the relatively large hippocampal-parahippocampal mask area (8315 voxels, Fig. 3B), the schizophrenia group showed ‘hotspots’ of reduced α5-GABA_A_R availability, mainly in anterior mTL (Fig. 6A), which overlapped with the areas of reduced theta power (Fig. 3C) and PLV (Fig. 4B) in schizophrenia subjects. The latter was unlikely to be due to chance: for the theta PLV group contrast, 106/146 voxels overlapped with the 1133 voxels showing reduced α5-GABA_A_R availability (permutation testing: *P *=* *0.013; Fig. 6B). In the theta power group contrast, 39/140 voxels overlapped (*P *=* *0.15). Cue-related change in PLV in the mTL area showing control on versus schizophrenia phase coupling differences (Fig. 4B) did not correlate significantly with α5-GABA_A_R availability in left hippocampus, however (*r* = −0.39, *P *=* *0.13), although there were only six controls and 11 subjects with schizophrenia who had both PET and MEG.

## Discussion

We have shown that mPFC-mTL dysconnectivity during memory retrieval in schizophrenia is characterized by a loss of theta phase coupling. Summarizing our findings, we observed reduced mTL theta power and mPFC-mTL theta phase coupling in subjects with schizophrenia versus controls, specific to the 1–8 Hz frequency band, not caused by antipsychotic medication and unrelated to cannabis use. Furthermore, schizophrenia subjects exhibited mild behavioural impairments in spatial memory performance, but more profound deficits in a hippocampal-dependent memory integration task. Notably, the mTL region showing reduced theta phase coupling in our MEG study coincided with areas of reduced α5-GABA_A_R availability in an overlapping schizophrenia sample (measured with PET).

### Hippocampal-prefrontal coupling in schizophrenia

This study demonstrates a disruption of oscillatory coupling in schizophrenia between mTL and the principle hippocampal-prefrontal projection zone (mPFC), primarily in the (1–8 Hz) theta band (Hedges' *g *=* *0.8).

One might question why the schizophrenia group did not show more marked impairments in the spatial memory task, given their loss of mPFC-mTL coupling during that task, and their profound impairment at the indirect inference in the memory integration task, which is known to depend on mPFC-mTL coupling. One reason may be that the indirect inference is very dependent on recurrent hippocampal circuitry (Horner *et al.*, 2015; Banino *et al.*, 2016), whereas neighbouring mTL areas (or even mid-cingulate cortex; Fig. 5) can contribute to spatial memory task performance. Indeed, epilepsy patients with hippocampal resections can still perform a similar spatial memory task (Vikbladh *et al.*, 2019). Interestingly, patients with hippocampal lesions are more impaired at precise positional memory (*d *>* *1) than general object-location memory (*d *<* *0.5) (Kessels *et al.*, 2001): a pattern shared by our schizophrenia group, who were under-represented in the highest centiles of accuracy (Fig. 1B).

Another reason for the schizophrenia performance differences between tasks may be that spatial tasks are more reliant on right hippocampus (Kessels *et al.*, 2001)—as the correlation of our controls’ performance with PLV in right mTL suggests—whereas the memory integration effect is strongest in left hippocampus (Backus *et al.*, 2016). Although hippocampal dysfunction is likely bilateral in schizophrenia subjects (Hanlon *et al.*, 2011), hippocampal pathology is greater on the left (Harrison, 2004). If mPFC-mTL communication is worse on the left in schizophrenia, as our results imply, this could also explain the performance differences in schizophrenia subjects on these tasks, and the lack of relationship between memory integration performance and mPFC-mTL coupling in the other task.

We had predicted that mPFC-mTL coupling would correlate with spatial memory performance in both groups: this relationship was only significant in controls, however. This may be because the schizophrenia group compensated by using other circuits to perform the task: we found some preliminary evidence of this, in that their performance correlated with cue-related theta power and PLV in a mid-cingulate control area. Controls did not seem to use this area to perform the task.

Consistent with our results, reduced hippocampal-mPFC functional connectivity is seen in resting state functional MRI data in subjects with schizophrenia (Zhou *et al.*, 2008), and hippocampal-prefrontal functional connectivity is reduced in antipsychotic-naïve first episode schizophrenia but normalizes following treatment (Blessing *et al.*, 2019). Also, a resting state MEG study found reduced right hippocampal coupling (in theta) to the rest of the medial temporal network in healthy participants with the schizophrenia risk alleles of the ZNF804A polymorphism (Cousijn *et al.*, 2015).

There is also a large literature on abnormal interactions between hippocampus and dorsolateral prefrontal cortex in schizophrenia. These areas display functional abnormalities during working memory tasks in schizophrenia subjects, high-risk populations, healthy participants with schizophrenia risk alleles, and animal models (Bähner and Meyer-Lindenberg, 2017). Indeed, subjects with the 22q11.2 microdeletion show abnormal hippocampal-frontoparietal network functional connectivity in resting state functional MRI (Schleifer *et al.*, 2019).

Numerous EEG studies have found task-related theta power loss in schizophrenia, e.g. during Go-No Go tasks (Simmonite *et al.*, 2015) and in oddball responses (Javitt *et al.*, 2018), which involve hippocampal-prefrontal areas in MEG (Recasens *et al.*, 2018). Reduced theta coupling has also been found in schizophrenia subjects, e.g. between fronto-temporal regions during talking (Ford *et al.*, 2002) and recognition memory (Thézé *et al.*, 2019), between fronto-parietal regions in a working memory task (Griesmayr *et al.*, 2014), within PFC during the continuous performance task (Ryman *et al.*, 2018), and between cingulate and sensorimotor cortex during the Stroop task (Popov *et al.*, 2019). Our findings advance this literature by demonstrating impaired theta coupling with a deep mTL source that is key to schizophrenia pathology and present in animal models, but which EEG cannot resolve.

Interestingly, resting state EEG studies often show increased theta coupling in schizophrenia (Andreou *et al.*, 2015; Di Lorenzo *et al.*, 2015; Gomez-Pilar *et al.*, 2018; Krukow *et al.*, 2018)—although not always (Tauscher *et al.*, 1998; Koenig *et al.*, 2001)—indicating that task-baseline theta connectivity differences in schizophrenia may be due to both an increased baseline and reduced effects of the task. We did not find increased theta power or coupling in mTL at baseline in schizophrenia subjects, however.

### Comparing schizophrenia results with animal studies

Our findings in subjects with schizophrenia recapitulate key aspects of the *Df*(*16*)*A^+/–^* mouse model: both show a loss of mTL-mPFC coupling during retrieval in the low end of the broader theta (1–8 Hz) range, rather than a broadband loss of coupling (as in the MIA model). Nevertheless, the MIA (and neonatal ventral hippocampal lesion) models suggest that antipsychotics may rescue hippocampal-mPFC theta coherence (Dickerson *et al.*, 2012; Lee *et al.*, 2014). Our underpowered comparison of schizophrenia ON and OFF antipsychotics was not statistically significant, but the numerical difference in coupling between these groups was in the same direction as that found in these animal models. The main difference between the *Df*(*16*)*A^+/–^* and schizophrenia results was that only the latter showed a loss of task-related theta power in mTL.

Hippocampal-mPFC theta coupling correlates with spatial memory performance in rodents (Jones and Wilson, 2005) and here we demonstrated this in humans for the first time: a previous attempt to do so may have lacked sufficient power (Kaplan *et al.*, 2014). The *Df(16)A^+/–^* mice are impaired in the task acquisition phase (Stark *et al.*, 2008), whereas schizophrenia subjects’ performance is less frequently at ceiling levels (similar to reward learning tasks in which performance differences between subjects with schizophrenia and controls are greatest at the easiest conditions, when controls perform at ceiling) (Gold *et al.*, 2012).

Other schizophrenia rodent models have also shown disturbances of hippocampal theta, e.g. in theta synchrony within hippocampus (Kaefer *et al.*, 2019), loss of theta phase coupling with PFC (Del Pino *et al.*, 2013), or in ‘hyper-coupling’ to PFC (Hartung *et al.*, 2016; Bygrave *et al.*, 2019), or decoupling during sleep (Phillips *et al.*, 2012), indicating theta dysfunction in this circuit may be common to many genetic and environmental risk factors for schizophrenia and other psychiatric disorders (Godsil *et al.*, 2013).

The co-localization of phase coupling loss with reduced α5-GABA_A_R availability in a wider schizophrenia sample warrants further investigation. One key question—if they are related—is whether α5-GABA_A_R reduction is pathological or compensatory. Theoretically it could be either: α5-GABA_A_Rs phasically inhibit somatostatin interneurons but also tonically inhibit pyramidal neurons, and blocking the latter in mice improves spatial memory (Magnin *et al.*, 2019). Marques *et al.* (2020) found that α5-GABA_A_R availability in unmedicated schizophrenia, while reduced overall, correlated positively with total symptoms (PANSS).

### Recognition and associative memory in schizophrenia

The schizophrenia group showed a striking impairment (Hedges' *g *=* *1.2) in indirect inference in the memory integration task, which may depend on hippocampal-mPFC theta coupling (Backus *et al.*, 2016), although we could not establish such a relationship across tasks. The indirect ‘A-C’ inference is impaired in chronic (Armstrong *et al.*, 2012*a*, *b*) and first episode schizophrenia (Armstrong *et al.*, 2018) (although our sample is the first IQ-matched comparison), and relational memory impairments in schizophrenia relate to hippocampal dysfunction (Ragland *et al.*, 2015). Other hippocampal-dependent cognitive operations, such as transitive inference, are also impaired in schizophrenia subjects and in unaffected relatives (Titone *et al.*, 2004; Onwuameze *et al.*, 2016) although not necessarily in early psychosis (Williams *et al.*, 2012).

The higher false alarm rate (but unchanged hit rate) in subjects with schizophrenia (Hedges' *g *=* *1.0) in the recognition memory test matches findings in recognition (Weiss *et al.*, 2004) and spatial working memory (Mayer and Park, 2012; Starc *et al.*, 2017). In a population sample performing an auditory perceptual task, false alarm rate correlated with auditory hallucinations (de Boer *et al.*, 2019). Specific increases in false alarms are predicted by disinhibition in neural network models (Murray *et al.*, 2014) and GABA blockade in PFC in rats selectively increases false alarms in an attentional task (Auger *et al.*, 2017) similar to schizophrenia (Demeter *et al.*, 2013).

### Limitations

We attempted to exclude any potential confounds of antipsychotic medication or cannabis use, and remove variance associated with linear effects of eye movements (on theta power) and both theta power and head movements (on theta phase coupling). Nevertheless, non-linear effects of these variables will remain. Furthermore, the hippocampus also contains nicotinic cholinergic receptors (nAChRs) (Dannenberg *et al.*, 2017), which can modulate theta power in hippocampal slices (Lu and Henderson, 2010) and following brainstem stimulation in anaesthetized mice (Stoiljkovic *et al.*, 2016). Thus it is possible—given schizophrenia subjects generally smoke more than controls, and that we asked subjects to refrain from smoking prior to testing—that the schizophrenia group might have had more nicotine-withdrawal effects than the control subjects, which may have contributed to group differences in theta power. Whether nAChRs can also modulate theta coupling is unknown, but future studies should attempt to counterbalance nicotine effects.

Volume conduction is also a concern in source-reconstructed data (e.g. whether ‘hippocampal’ theta actually originates in PFC), but this paradigm and source localization using both MEG and functional MRI in the same subjects confirmed the localization of hippocampal activity (Kaplan *et al.*, 2012). Increases in hippocampal theta power in this paradigm have also been seen using intracranial EEG (Bush *et al.*, 2017).

The numbers of subjects with both PET and MEG imaging was small: the exploratory finding of overlap in areas of reduced α5-GABA_A_R availability and reduced PLV in mTL in schizophrenia needs to be replicated in a larger sample of subjects who have all had both MEG and PET investigations. It is also unclear whether the relationship between these findings is causal.

## Conclusion

We have shown that mPFC-mTL dysconnectivity during memory retrieval in schizophrenia is characterized by a loss of theta (1–8 Hz) phase coupling. This coupling loss in schizophrenia was not explained by the co-existing loss of mTL theta power, head movements, antipsychotic medication or cannabis use. The schizophrenia group were also impaired at two memory tasks involving mPFC-mTL interaction, although more work is needed to understand the relationships between performance and theta coupling in schizophrenia. These findings are important as they were predicted by a genetic schizophrenia mouse model, which could be used for translational studies of pharmacological targets that modulate theta and thus cognition. As the site of coupling differences overlapped with areas of reduced α5-GABA_A_R availability in a larger schizophrenia sample, and as these receptors participate in the generation of theta oscillations, the relationship between them warrants further investigation.

## Supplementary Material

awaa035_Supplementary_Data

## Abbreviations

MEG =: magnetoencephalography
mPFC =: medial prefrontal cortex
mTL =: medial temporal lobe
PANSS =: Positive and Negative Symptoms Scale
PLV =: phase locking value
