# Supplementary material for: Impaired theta phase coupling underlies frontotemporal dysconnectivity in schizophrenia
Source: Brain. 2020 Apr 1;143(4):1261–77. doi: 10.1093/brain/awaa035 (PMC7174039; doi:10.1093/brain/awaa035)
Supplement: awaa035_Supplementary_Data [file awaa035_supplementary_data.pdf]

Impaired theta phase coupling underlies frontotemporal dysconnectivity in schizophrenia: Supplementary material

Supplementary tables

No.	Age (yrs)	Diag	Medication (daily dose)	THC+	PANSS-P	PANSS-N	PANSS-G	PET-MEG (days)
1	30	SCZ	None	0	23	19	30	1
2	21	SCZ	Risperidone 0.5 mg	0	9	7	23	-
3	60	SCZ	Olanzapine 10 mg	1	8	11	22	5
4	41	SCZ (FEP)	Sulpiride 700 mg	1	-	-	-	25
5	35	SCZ	Risperidone [50mg depot two weekly]	0	15	15	23	8
6	24	SCZ (FEP)	None	0	11	9	24	14
7	30	SCZ	Olanzapine 20 mg	1	14	19	24	36
8	39	SCZ (FEP)	None	1	23	31	47	2
9	26	SCZ (FEP)	None	1	8	11	25	-
10	36	SCZ	Olanzapine 5 mg	0	7	9	17	-
11	21	SCZ (FEP)	None	1	8	15	19	35
12	24	SCZ (FEP)	Aripiprazole 20 mg	0	7	7	27	-
13	26	SCZ	Paliperidone [90mg depot four weekly]	1	15	16	30	-
14	29	SCZ (FEP)	Olanzapine 20 mg	0	-	-	-	-
15	39	SCZ (FEP)	Risperidone 2 mg	1	7	9	25	-
16	39	SCZ (FEP)	None	0	-	-	-	16
17	20	SCZ (FEP)	None	1	9	19	33	25
18	24	SCZ (FEP)	None	0	9	8	19	85
			10 medicated, 8 unmedicated	9+ 9-	11.5 ±5.4	13.7 ±6.5	25.9 ±7.3	22.9 ±24.0

Table S1: Detailed characteristics of the Scz group (FEP = first episode psychosis)

	**Age (yrs)**	**Sex (M/F)**	**Handed (R/L)**	**Education (years)**	**IQ (WTAR)**	**Digit span - Fwd**	**Digit span - Bkwd**
Controls (n=26)	31.5 ±10.1	19 M<br>7 F	22 R<br>4 L	15.9 ±1.1	98.7 ±5.8	9.0 ±2.3	6.8 ±2.1
Scz (n=18)	31.3 ±10.0	15 M<br>3 F	16 R<br>2 L	14.2 ±2.2	98.9 ±10.6	7.9 ±2.2	5.4 ±2.3
Statistical difference	p=1<br>t=0.05	p=0.7<br>χ^2=0.2	p=1<br>χ^2=0.2	**p=0.003**<br>**t=3.3**	p=0.9<br>t=-0.08	p=0.13<br>Z=1.5	**p=0.03**<br>**Z=2.2**

Table S2: Characteristics of control and Scz groups (mean ±std)

Supplementary Results

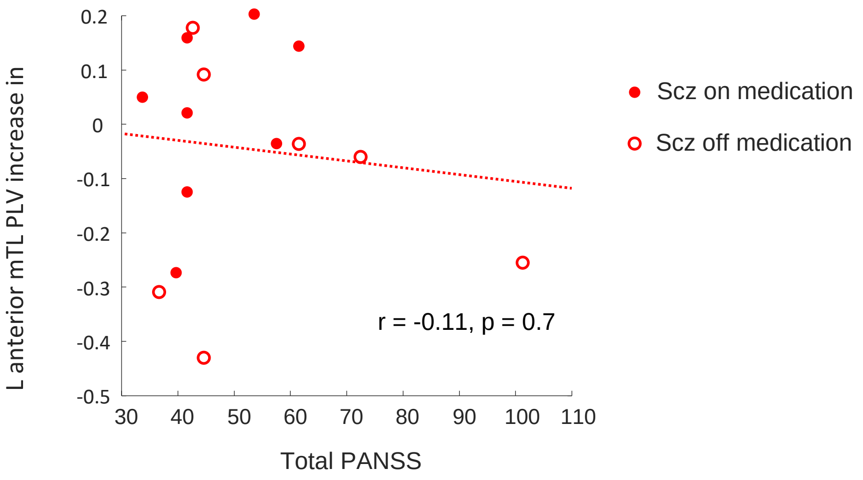

Supplementary Figure 1: Absence of correlation between left anterior mTL PLV change and schizophrenia symptoms

This figure plots cue-related left anterior mTL PLV change against current symptoms of schizophrenia assessed using the Positive and Negative Symptom Scale (PANSS) in 15 Scz participants (three did not have PANSS measurement). There does not appear to be a relationship between symptom severity and PLV change.

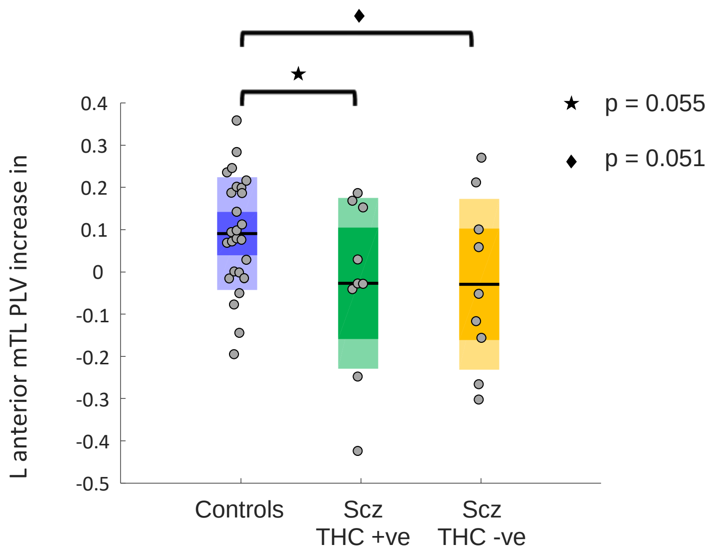

Supplementary Figure 2: Effects of cannabis use on left anterior mTL PLV change in Scz

This figure plots the same data in the same format as Figure 4C, but splits the Scz group into those who tested positive for cannabis use (THC +ve) and those who did not (THC -ve). The figure shows that cannabis use is unlikely to be driving the Scz group's loss of PLV, because the distributions of PLV in Scz testing positive vs negative for cannabis are almost identical (t_{16}=0.02, p=1).
